# Survival outcomes analysis according to mismatch repair status in locally advanced rectal cancer patients treated with neoadjuvant chemoradiotherapy

**DOI:** 10.3389/fonc.2022.920916

**Published:** 2022-08-08

**Authors:** Lin Chen, Xudong Yang, Yuanyuan Zhang, Jie Liu, Qixin Jiang, Fang Ji, Jinli Gao, Zhuqing Zhou, Hao Wang, Jun Huang, Chuangang Fu

**Affiliations:** ^1^ Department of Colorectal Surgery, Department of General Surgery, Shanghai East Hospital, School of Medicine, Tongji University, Shanghai, China; ^2^ Department of Pathology, Shanghai East Hospital, School of Medicine, Tongji University, Shanghai, China; ^3^ Department of Colorectal Surgery, Chang hai Hospital, Naval Medical University, Shanghai, China; ^4^ Department of Colorectal Surgery, The 6^th^ Affiliated Hospital of Sun Yat-sen University, Guangzhou, China

**Keywords:** mismatch repair status, survival outcome, locally advanced rectal cancer, neoadjuvant chemoradiotherapy, retrospective cohort study

## Abstract

**Background:**

The predictive role of mismatch repair (MMR) status for survival outcomes and sensitivity in neoadjuvant chemoradiotherapy settings for patients with locally advanced rectal cancer (LARC) has been inconclusive.

**Methods:**

A retrospective cohort of patients with LARC treated with neoadjuvant chemoradiotherapy (nCRT) was recruited. After adjusting for baseline characteristics, we used propensity score matching to reduce the effect of potential confounding factors on MMR status. The primary analysis was based on overall survival as the more important endpoint.

**Results:**

This study included 269 patients. Patients with defective MMR (dMMR) were younger (58.5% vs. 60.0%, p=0.0274) and had lower body mass indices (p=0.0091), higher differentiation grades (p=0.0889), and more advanced rectal cancers (clinical T4 or T4b, p=0.0851; M1, p=0.0055) than those with proficient MMR (pMMR). However, propensity score-matched patients with dMMR (p=0.0013) exhibited superior overall survival, even in the M1 subgroup. More importantly, patients with proficient MMR who undergo early pathological downstaging, especially lymph node pathological downstaging, can achieve a prognosis similar to that of patients with dMMR.

**Conclusion:**

The clinical significance of this retrospective study mainly includes two points: (1) Data from our study confirmed that LARC patients with dMMR status had better overall survival rates after nCRT, even in the M1 subgroup. (2) Similar survival outcomes were observed in older and female patients with early lymph node pathological downstaging, regardless of dMMR or pMMR.

## Introduction

Colorectal cancer is the second most common cancer worldwide, accounting for 9.4% of all cases, including an estimated 732,210 rectal cancer cases reported in 2020 (1). For locally advanced rectal cancer (LARC) patients with clinical T3 or T4 tumors or positive lymph node(s), neoadjuvant chemoradiotherapy (nCRT) is recommended to achieve tumor downstaging and local disease control, and possibly sphincter preservation; radical surgical resection can be performed after nCRT is completed (2). For patients with unresectable LARC, preoperative concurrent chemoradiotherapy is currently the standard treatment (2), but some of them eventually become resistant to chemoradiotherapy, thus necessitating better early efficacy.

The etiology of colorectal cancer involves multiple genes and chromosomal instability, microsatellite instability due to mismatch repair (MMR) deficiency, and epigenomic instability (2). Currently, less than two-thirds of LARC patients benefit from nCRT (3), and microsatellite instability (MSI) may be a relevant influencing factor. MSI refers to the hypermutator phenotype secondary to frequent polymorphisms in short repetitive DNA sequences and single nucleotide substitutions, as a consequence of DNA MMR deficiency (4). Approximately 5–10%of rectal cancers are characterized by MSI, reflecting inactivation of MMR genes (5, 6)or defective MMR (dMMR). Nicolas et al. (7) reported that dMMR patients in the nCRT setting had longer recurrence-free survival (RFS) (p<0.0001) and improved but non-significant overall survival (OS). Conversely, in a recent study by Ye et al (8), in LARC patients treated with nCRT, dMMR status was found to be associated with a numerically non-significant trend in a shorter disease-free survival (DFS) (hazard ratio [HR] and 95% confidence interval [CI]:1.50[0.92, 2.44], p=0.108) when compared with proficient MMR (pMMR) system status.

In the nCRT setting, these apparently inconsistent findings regarding better defined DFS and OS based on MMR status, and the lack of solid evidence in the survival outcome warrant further investigation. The most important prognostic factors for OS include the clinical and pathologic extent of disease (TNM stage), pathologic type, and circumferential resection margin (2). With these key study data available, we conducted this retrospective multicenter cohort study in China to investigate survival outcomes and explore the role of MMR status in patients with LARC initially treated with nCRT.

## Patients and methods

### Patients

In this retrospective cohort study, patients with LARC treated with nCRT were recruited from three study sites in Shanghai and Guangzhou, China. Patients were eligible for inclusion in the study if they were 18 years of age or older, had been diagnosed with a malignant tumor but no Lynch syndrome by surgery/biopsy or cytological pathology, had undergone radical rectal cancer surgery after nCRT, had a pathological report with well-defined tumor regression grades (TRGs), obtained relevant clinical follow-up data, and were immunohistochemically detected with MMR or MSI status. This study was approved by the Ethics Committees of Shanghai East Hospital, Tongji University School of Medicine (No.2020-148) and was conducted in accordance with the Declaration of Helsinki of the World Medical Association. The requirement for written informed consent from each participating patient was waived in the retrospective analysis.

Patients in the study received nCRT with a median of 25 radiotherapy fractions (interquartile range [IQR] =25–25) and chemotherapy with capecitabine-containing regimens used for all but one patient. Patients received a mean dose of 50.4 Gy over 5 weeks. After an observation period of 8–12 weeks, almost all the patients underwent radical surgery. All patients underwent R0 resection, except for three patients with dMMR who underwent R1 resection. Subsequent adjuvant chemotherapy and salvage chemotherapy were performed according to the National Comprehensive Cancer Network (NCCN) guidelines. We evaluated the early efficacy of nCRT in patients with LARC using the TRG system (NCCN criteria) to correspond to pathological complete response (TRG=0), partial response (TRG=1), stable disease (TRG=2), and disease progression (TRG=3).

The clinical response to nCRT treatment were assessed using the RECIST criteria (9), and patients followed every 3 months within the first two years and every 6 months thereafter until the fifth year. According to the criterion-based results, RFS per patient was calculated from the date nCRT therapy was initiated to the date of either tumor progression or recurrence, whichever occurred first. OS was defined as the time from the initiation of nCRT to death from any cause.

### Determination of MMR status

Immunohistochemistry (IHC) staining for MMR proteins was performed on all tumor specimens. Most of the tumors were subjected to IHC staining after neoadjuvant treatment, whereas patients with TRG=0 were tested using biopsy samples available before neoadjuvant treatment. ZSGB-BioSolutions SPlink Detection Kits (Zhong Shan Jinqiao, Beijing, China) and an automated IHC/ISH slide staining instrument (The BenchMark XT platform)were used to stain the formalin-fixed, paraffin-embedded, 3-μm sections according to the manufacturer’s instructions.

IHC staining was performed using diagnostic antibodies against MLH1 (clone ES05; Zhong Shan Jinqiao, Beijing, China, 1:40),MSH2 (clone RED2; Zhong Shan Jinqiao, Beijing, China, 1:200),MSH6 (clone UMAB258; Zhong Shan Jinqiao, Beijing, China,1:200), and PMS2 (clone EP51; Zhong Shan Jinqiao, Beijing, China, 1:40). When nuclear staining was absent from any tumor cells, but was present in normal epithelial and stromal cells, protein expression was defined as absent (loss or abnormal). The MMR status of all patients was determined based on immunohistochemical analysis of the expression of the four aforementioned MMR proteins, whereas the dMMR status was defined as the loss of expression of one or more of these proteins.

In our study, 30 dMMR tumors showed loss of MMR protein expression: MLH1 (n=4, 13.3%), MSH2 (n=13, 43.3%), MSH6 (n=14, 46.7%), and PMS2 (n=10, 33.3%). The loss of MLH1 expression completely overlapped with that of PMS2 expression, while only one patient showed overlap between MLH1, MSH2, and PMS2 expression loss (**Supplementary Table 1** and **Supplementary Figure 1**).

### Statistical analysis

All study data were analyzed using SAS version 9.4 (SAS Institute, Cary, NC, USA). Two-sided p values of less than 0.05 were considered statistically significant. In the cohort study, the demographic and clinicopathological characteristics were summarized according to MMR status. Pearson’s chi-squared test or Fisher’s exact test was used for categorical data, and either a t-test or Wilcoxon rank-sum test was used for continuous data, as appropriate. Multiple logistic regression modeling was used to explore potential clinical factors associated with tumor regression grade immediately after nCRT. Survival curves were constructed using the Kaplan–Meier method, and survival data were statistically analyzed using the log-rank test.

To reduce the effect of potential confounding with MMR status, we adjusted for baseline characteristics between the two MMR status groups using propensity score matching. The propensity score represents the probability of dMMR status conditional on the measured clinical variables at baseline. After variable selection, the propensity score was calculated for each patient using a logistic regression model with age, sex, body weight, body mass index, degree of tumor differentiation, clinical T stage, clinical N stage, and clinical M stage as variables. The same statistical methods were used for the analysis of patient data from the propensity-score-matched cohort.

## Results

### Demographic and clinical characteristics

From January 2011toAugust 2020,428 patients underwent nCRT. Subject to the testing conditions available, a total of269 patients were determined to have dMMR or pMMR status based on their IHC findings using histological specimens available at the time of radical surgery (**Supplementary Figure 2**). The median follow-up time of all patients was 20.0 (IQR = 11.0 - 35.0) months in the whole study.

The relevant characteristics of the patients at the initiation of nCRT are summarized in **Table 1**. The median age of all included patients with dMMR or pMMR status was 58.5vs. 60.0 years, respectively. Patients with dMMR tumors were younger (p=0.0274), had lower body mass indices (p=0.0091), and were more likely to be diagnosed with a higher differentiation grade (middle-high or high:10.0% vs. 2.1%, p=0.0889) and later phases of rectal cancer (clinical T4 or T4b, p=0.0851; M1, p=0.0055) than those with pMMR tumors. The type of adenocarcinoma was dominant (96.7%vs.94.1%) between the two MMR status groups. Overall, the majority of the key patient characteristics differed considerably between the two MMR status groups.

**Table 1:** Clinical characteristics of 269 MMR patients.

	dMMR (N=30)	pMMR (N=239)	P values
Age at diagnosis (yrs),median(IQR)	58.5 (41.0, 65.0)	60.0 (51.0, 67.0)	0.0274
Male sex, n (%)	25 (83.3%)	179 (74.9%)	0.3722
Weight(kg), mean (SD)	60.4 (11.10)	64.3 (10.6)	0.0591
Body mass index(kg/m2), mean (SD)	21.3 (3.43)	23.1 (3.45)	0.0091
Differentiation, n (%)			0.0889
Low	3 (10.0%)	16 (6.7%)	
Low-middle	1 (3.3%)	19 (7.9%)	
Middle	23 (76.7%)	199 (83.3%)	
Middle-high	1 (3.3%)	1 (0.4%)	
High	2 (6.7%)	4 (1.7%)	
Distance from anus to tumor margin(cm), mean (SD)	4.5 (2.51)	5.0 (2.49)	0.2986
Max diameters of tumor(cm), mean (SD)	3.2 (1.84)	2.9 (1.40)	0.3168
CEA before surgery (ng/mL),median(IQR)	2.6 (2.0, 3.8)	2.8 (1.9, 4.8)	0.5942
CA19-9 before surgery (U/mL),median(IQR)	6.5 (3.5, 15.8)	9.5 (4.5, 16.5)	0.1758
Pathologic type, n (%)			0.5784
Tubular	1 (3.3%)	3 (1.3%)	
Squamous	0	1 (0.4%)	
Adenocarcinoma	29 (96.7%)	225 (94.1%)	
Mucinous	0	10 (4.1%)	
cT stage, n (%)			0.0851
2	5 (16.7%)	31 (13.0%)	
2b	0	1 (0.4%)	
3	17 (56.7%)	177 (74.1%)	
3b	0	1 (0.4%)	
4	8 (26.7%)	22 (9.2%)	
4b	0	7 (2.9%)	
cN stage, n (%)			0.3423
0	8 (26.7%)	53 (22.3%)	
1	10 (33.3%)	112 (47.1%)	
2	12 (40.0%)	73 (30.7%)	
Missing	0	1	
cM stage, n (%)			0.0055
0	23 (76.7%)	224 (93.7%)	
1	7 (23.3%)	15 (6.3%)	

CA, carbohydrate antigen; CEA, carcinoembryonic antigen; dMMR, defective mismatch repair system; IQR, interquartile range; M, metastasis; pMMR, proficient mismatch repair system; SD, standard deviation.

### Association of MMR status with downstaging and survival data

**Figure 1:**
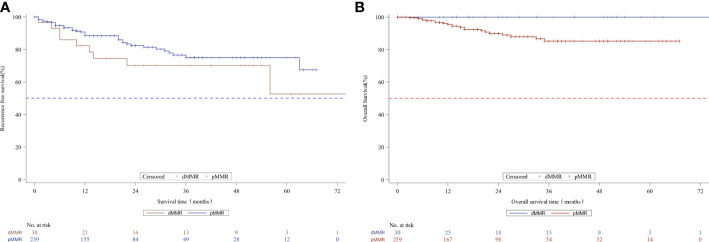
Kaplan-Meier curves for recurrence-free survival **(A)** and overall survival **(B)** according to MMR status in the whole unmatched patients. MMR denotes mismatch repair, dMMRdefective MMR; pMMRproficientMMR.

We analyzed the association between MMR status and downstaging and survival outcomes. A total of 269 patients were eligible for outcome comparison. The tumor downstaging rate after nCRT did not differ between the two MMR status groups, and there was no significant difference in the pathological complete response (pCR) rate following nCRT with dMMR versus the pMMR population (p>0.05, **Table 2**). Patients with dMMR showed a numerically worse RFS rate (p>0.05, **Table 2**; **Figure 1**), but a numerically better OS profile (p>0.05, **Table 2**; **Figure 1**) than those with pMMR.

**Table 2:** Downstaging, recurrence and overall survival endpoints of the 269 MMR patients.

	MMR Status		
	dMMR (N=30)	pMMR (N=239)	p-value
Downstaging, n (%)			
TRG 0 + 1*	11 (36.7%)	82 (34.3%)	0.7981
TRG 0*	3 (10.0%)	26 (10.9%)	0.8837
Recurrence status			
Yes, n (%)	9 (30.0%)	38 (15.9%)	0.0552
6-month RFS rate (95%CI, %)	85.9 (66.7, 94.5)	94.8 (91.0, 97.0)	0.2094
1-year RFS rate (95%CI, %)	82.4 (62.7, 92.3)	88.4 (83.1, 92.1)	
2-year RFS rate (95%CI, %)	70.1 (48.8, 83.9)	82.4 (75.5, 87.5)	
3-year RFS rate (95%CI, %)	70.1 (48.8, 83.9)	75.0 (66.1, 81.9)	
5-year RFS rate (95%CI, %)	52.6 (18.6, 78.2)	75.0 (66.1, 81.9)	
Death status			
Alive, n (%)	30 (100%)	218 (91.2%)	0.1438
Median OS time (95%CI)	NC (NC to NC)	NC (NC to NC)	0.0705
2-year OS rate (95%CI, %)	100 (100, 100)	89.9 (84.1, 93.7)	
5-year OS rate (95%CI, %)	100 (100, 100)	85.2 (77.4, 90.4)	

CI, confidence interval; dMMR, defective mismatch repair system; NC, not calculated; OS, overall survival; pMMR, proficient mismatch repair system; RFS, recurrence-free survival; TRG, tumor regression grade.

* TRG=0, pathological complete response; TRG=1, partial response; TRG=2, stable disease; TRG=3, disease progression.

Given the potential prognostic value of TRG for survival in rectal cancer, we further analyzed the related baseline characteristics. TRG or early downstaging after nCRT (TRG=0 or TRG=0+1) was found to be associated with patients’ sex (odds ratio=0.49, 95%CI = 0.27-0.90 vs. female, p=0.0218) or age (odds ratio=1.04, 95%CI = 1.00–1.08, p =0.0489), instead of MMR status (p>0.05) (**Table 3**). Women or older patients were more likely to achieve TRG downstaging than men or young patients.

**Table 3:** Characteristics at baseline associatedwith downstaging (TRG=0 or TRG=0 or 1) among the 269 MMR patients according to multiple logistic regression.

	TRG =0 as downstaging*		TRG =0, 1as downstaging*	
	OR (95% CI)	p-value	OR (95% CI)	p-value
MMR status (vs. pMMR)	1.10 (0.28,4.31)	0.8862	1.40 (0.60,3.29)	0.4334
Sex (vs. Female)	0.47 (0.19,1.13)	0.0927	**0.49 (0.27,0.90)**	**0.0218**
Age, per one year of increase	**1.04 (1.00,1.08)**	**0.0489**	1.02 (0.99,1.04)	0.1405
BMI, per one unit of increase	0.93 (0.83,1.06)	0.2707	1.01 (0.94,1.09)	0.7868
Distance from anus to tumor margin, per one cm of increase	0.96 (0.80,1.16)	0.6940	1.04 (0.94,1.16)	0.4321
Differentiation<σπ>^Δ^</σπ> (low vs. high)	1.44 (0.49,4.28)	0.5120	1.03 (0.49,2.20)	0.9310
cT stage (T3+4 vs. 2)	0.67 (0.22,2.05)	0.4822	0.56 (0.26,1.22)	0.1434
cN stage (N1+2 vs. 0)	0.66 (0.26,1.64)	0.3665	0.82 (0.43,1.56)	0.5472
cM stage (M1 vs. 0)	0.29 (0.03,2.49)	0.2595	0.68 (0.24,1.93)	0.4707

BMI, body mass index (kg/m2); CI, confidence interval; dMMR, defective mismatch repair system; NC, not calculated; OR, odds ratio; pMMR, proficient mismatch repair system; TRG, tumor regression grade.

* TRG=0, pathological complete response; TRG=1, partial response; TRG=2, stable disease; TRG=3, disease progression.

<σπ>^Δ^</σπ> Low differentiation denotes low or low-middle grades of tumor cells, whereas high differentiation denotes middle,middle-high, or high differentiation. Bold values, p-values of less than 0.05.

### Propensity score-matched cohort

**Figure 2:**
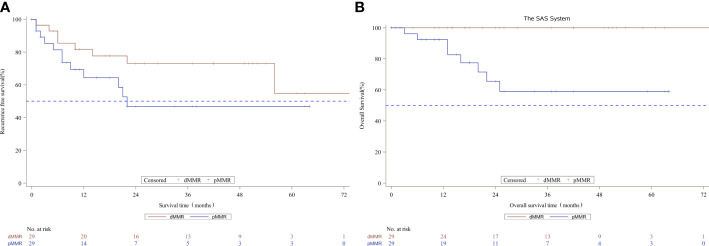
Kaplan-Meier curves for recurrence-free survival **(A)** and overall survival **(B)** according to MMR status in the propensity score matched subset patients. MMR denotes mismatch repair, dMMRdefective MMR; pMMRproficientMMR.

After propensity score matching, 29 of 239 pMMR patients (12.1%) were matched successfully with 29out of 30 dMMR patients. The distribution of propensity scores is not presented. Patients were well-matched in a patient number ratio of 1:1, and the two matched MMR groups were balanced with regard to all baseline characteristics (**Supplementary Table 2**). Kaplan-Meier survival curves after propensity score matching are shown in **Figure 2**. Favorable OS data were observed in matched patients with dMMR tumors compared to those with pMMR tumors (p=0.0013, log-rank test, **Table 4**),while a similar RFS rate was observed for patients with dMMR tumors(p=0.1371, log-rank test, **Table 4**). In this study, all 29 matched patients with dMMR tumors were still alive, with a 5-year survival rate of 100%,while 59.0% were reported in the pMMR tumors, even with a comparable number of M1 patients (n=6 vs. 7).

**Table 4:** Downstaging, recurrence and overall survival endpoints of the 58 matched MMR patients.

	MMR Status		
	dMMR(N=29)	pMMR(N=29)	p-value
Downstaging, n (%)			
TRG 0 + 1*	10 (34.5%)	7 (24.1%)	0.5648
TRG 0*	3 (10.3%)	2 (6.9%)	1.0000
Recurrence status			
Yes, n (%)	8 (27.6%)	12 (41.4%)	0.2692
6-month RFS rate (95%CI, %)	85.4 (65.7, 94.3)	81.4 (60.9, 91.8)	0.1371
1-year RFS rate (95%CI, %)	81.7 (61.5, 92.0)	64.4 (42.2, 79.8)	
2-year RFS rate (95%CI, %)	73.1 (51.4, 86.2)	46.8 (24.5, 66.3)	
3-year RFS rate (95%CI, %)	73.1 (51.4, 86.2)	46.8 (24.5, 66.3)	
5-year RFS rate (95%CI, %)	54.8 (18.9, 80.5)	46.8 (24.5, 66.3)	
Death status			
Alive, n (%)	29 (100%)	21 (72.4%)	0.0045
Median OS time (95%CI), months	NC (NC to NC)	NC (20 to NC)	0.0013
2-year OS rate (95%CI, %)	100 (100, 100)	65.5 (40.1, 82.2)	
5-year OS rate (95%CI, %)	100 (100, 100)	59.0 (33.4, 77.5)	

CI, confidence interval; dMMR, defective mismatch repair system; NC, not calculated; OS, overall survival; pMMR, proficient mismatch repair system; RFS, recurrence-free survival; TRG, tumor regression grade.

* TRG=0, pathological complete response; TRG=1, partial response; TRG=2, stable disease; TRG=3, disease progression.

### Survival outcome with downstage patients

**Figure 3:**
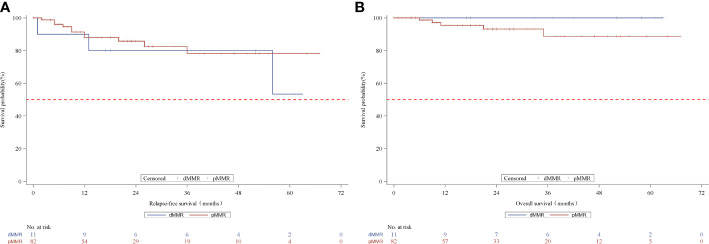
Kaplan-Meier curves for recurrence-free survival **(A)** and overall survival **(B)** according to MMR status in the TRG downstage subset patients. MMR denotes mismatch repair, dMMRdefective MMR; pMMRproficientMMR; TRG, tumor regression grade.

We further analyzed the survival outcomes in patients who achieved TRG downstaging (TRG=0 or 1) after nCRT. A similar 3-year RFS rate (80.0% vs. 78.2%, p=0.5661) and 3-year OS rate (100% vs. 88.7%, p=0.3515, **Figure 3**) based on MMR status were observed in patients with downstaged LARC.

## Discussion

To the best of our knowledge, the predictive role of dMMR in the OS efficacy of nCRT in patients with LARC remains inconclusive. However, our study findings showed that dMMR tumors were significantly associated with better OS rates among propensity score-matched patients.

In the adjuvant setting, colorectal cancer with MSI as reported in the available meta-analysis, have a significantly better prognosis than those with intact mismatch repair and may serve as a screening tool for detecting prognostic markers in LARC patient outcome and predictive markers for response to chemotherapy and immunotherapy (5, 10). Univariate and multivariate analyses showed that patients who were non-responsive to nCRT had worse OS than those responsive to nCRT(5-year OS rate:67% vs. 27%; median OS:3.1 vs. 8.3 years, p < 0.001; HR = 3.22, 95% CI = 1.68–6.20) (11).

Our study found that the dMMR status was not associated with a lower RFS rate. The high recurrence rate of 30% specified distant and local recurrences, the relatively advanced stage of LARC with dMMR versus pMMR at the beginning of nCRT (clinical T4:26.7% vs. 9.2%; M1:23.3% vs. 6.3%). TNM stage, especially T4, is likely more important as a prognostic factor than tumor regression (12). Nevertheless, similar TRG rates were gradually caught up after nCRT in our study, and the improvement might build a solid foundation for good subsequent OS despite an R0 resection rate of 90% with dMMR instead of 100% with pMMR. However, one possible explanation involves different chemotherapy regimens, such as the limited benefits of fluoropyrimidines (13). Preoperative consolidation chemotherapy with Capeox or mFOLFOX6 after nCRT can significantly improve DFS (14, 15). In the present study, a capecitabine-containing regimen was used for nCRT and adjuvant chemotherapy (capecitabine based CT).

Several studies (12, 16, 17) have shown that early TRG downstaging can predict patient prognosis with comparable performance for OS and DFS. Based on these data, we attempted to identify the clinicopathological characteristics at baseline that are likely to be associated with tumor downstaging. Older and female patients were more likely to achieve downstaging in our study (both p<0.05). The results should be noted that nodal downstaging is more valuable than tumor downstaging for predicting long-term survival (18). Therefore, patients with pMMR can still benefit from neoadjuvant chemoradiotherapy, as long as early downstaging can be achieved.

Furthermore, a recent phase 2 study (n=34) (19) demonstrated a very promising pCR rate after neoadjuvant immunotherapy with PD-1 blockade therapy (88% in the toripalimab plus celecoxib group vs. 65% in the toripalimab monotherapy group). The overwhelming early initial efficacy of immunotherapy indicated that it might be a potential therapeutic option for the LARC patients with dMMR and partial responsive patients with pMMR. A recent study showed that single-agent PD-1 blockade could even avoid surgery in patients with dMMR, and Watch & Wait strategy after PD-1 blockade was also safe in these patients (20). However, the unproportionally higher pCR rate with neoadjuvant PD-1 therapy in dMMR patients conveys huge unmet medical needs for pMMR tumors because it accounts for approximately 90–95% of LARC patients. Accordingly, we further analyzed the survival outcomes of patients who achieved pCR (TRG=0) downstaging after nCRT. Similar survival rates were observed in patients with dMMR and those with pMMR.

Despite these statistically conclusive findings, our cohort study has several limitations. First, the dMMR group was small. There are two main reasons for this: on the one hand, it occurs in less than 5% of rectal cancer patients compared with other carcinomas. However, because immunotherapy is increasingly recommended for neoadjuvant clinical trials, the number of cases available for retrospective studies is even smaller. In this study, we collected data from three sites in Shanghai and Guangzhou, China, to expand the study size. We included patients with metastatic dMMR for prognosis evaluation to explore whether the prognosis of the dMMR population in patients with advanced rectal cancer is still better than that of the pMMR population. We also used the propensity score matching method to reduce potential confounding factors with MMR status, including non-limited metastatic status as potential confounders. Second, we did not follow up the study patients sufficiently to obtain mature OS data. In addition, we did not consider genetic alterations coexisting with MMR gene mutations and the possible interactions between them, although their prevalence has been reported.

In summary, our retrospective analyses demonstrated that the dMMR status predicted better prognosis and OS rates in all patients with LARC after nCRT, even in the M1 subgroup. Similar survival outcomes were observed in patients with early lymph node pathological downstaging regardless of dMMR or pMMR status in older and female populations. Neoadjuvant chemoradiotherapy remains the effective treatment for patients with pMMR, for its impact on local tumor control and possible sphincter preservation even if no survival advantage has been reported.

## Data availability statement

The original contributions presented in the study are included in the article/**Supplementary Material**. Further inquiries can be directed to the corresponding authors.

## Ethics statement

The studies involving human participants were reviewed and approved by Shanghai East Hospital, Tongji University School of Medicine (No.2020-148). Written informed consent for participation was not required for this study in accordance with the national legislation and the institutional requirements. Written informed consent was not obtained from the individual(s) for the publication of any potentially identifiable images or data included in this article.

## Author contributions

LC, XY, and YZ conceived of and designed the study. JL wrote the Data Analysis section. ZZ bears the overall responsibility for the design, ethical conduct, and publication of the study. Administrative, technical, and material support were provided by HW. All authors were involved in the protocol discussion and took responsibility for data collection and verification. All authors have edited the draft, contributed substantially to the manuscript, and approved this submission.

## Funding

This work was supported by grants from the National Natural Science Foundation of China (No. 81773275, No. 81573004, N0. 81972885) and the Top-level Clinical Discipline Project of Shanghai Pudong (NO. PWYgf2018-04), Pudong New District Health and Family Planning Commission Youth Science and Technology Project (No. PW2016B-4), and Shanghai Health and Family Planning Commission Youth Science and Technology Project (No. 202040303), the National Key Clinical Discipline and the 1010 project of the 6th Affiliated Hospital of Sun Yat-sen University [1010CG (2020)-20].

## Conflict of interest

The authors declare that the research was conducted in the absence of any commercial or financial relationships that could be construed as a potential conflict of interest.

## Publisher’s note

All claims expressed in this article are solely those of the authors and do not necessarily represent those of their affiliated organizations, or those of the publisher, the editors and the reviewers. Any product that may be evaluated in this article, or claim that may be made by its manufacturer, is not guaranteed or endorsed by the publisher.

## Supplementary material

Supplementary Figure 1 Immunohistochemistry plots of MMR. **(A)** MLH1, **(B)** MSH2, **(C)** MSH6, and (D)PMS2.

Supp Figure 2 Patient flow chart. MMR, mismatch repair; dMMRdefective MMR, LARC locally advanced rectal cancer; pMMRproficientMMR; PS, propensity score.
