# Supplementary material for: Survival outcomes analysis according to mismatch repair status in locally advanced rectal cancer patients treated with neoadjuvant chemoradiotherapy
Source: Front Oncol. 2022 Aug 8;12:920916. doi: 10.3389/fonc.2022.920916 (PMC9393758; doi:10.3389/fonc.2022.920916)
Supplement: Supplementary Figure 1 — Immunohistochemistry plots of MMR. (A) MLH1, (B) MSH2, (C) MSH6, and (D)PMS2. [file Presentation_1.pptx]

## Slide 1
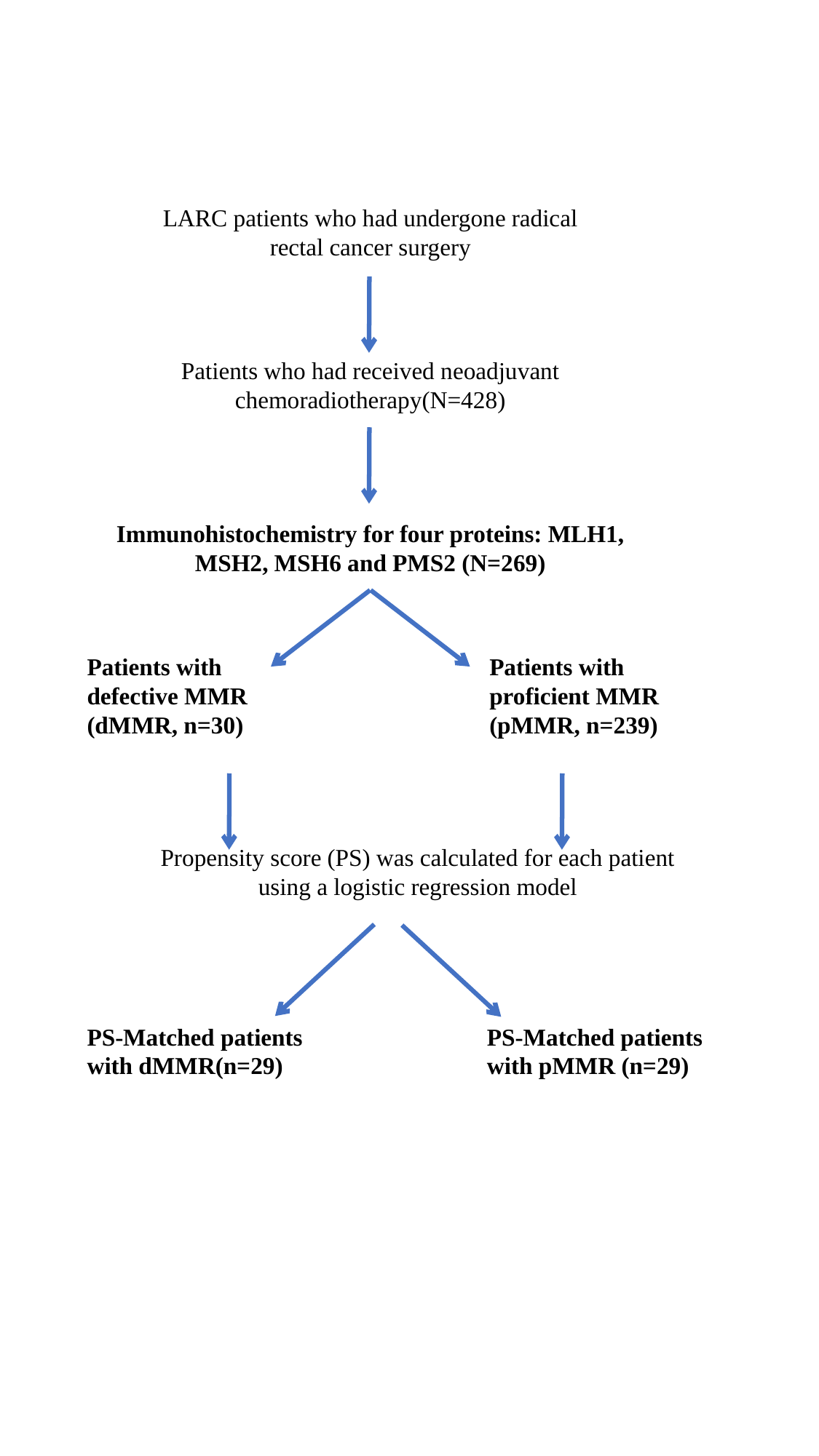

LARC patients who had undergone radical rectal cancer surgery
Recurrence free and overall survival (OS) for matched MMR patients
Patients who had received neoadjuvant chemoradiotherapy(N=428)
Immunohistochemistry for four proteins: MLH1, MSH2, MSH6 and PMS2 (N=269)
Patients with defective MMR (dMMR, n=30)
Patients with proficient MMR (pMMR, n=239)
Propensity score (PS) was calculated for each patient using a logistic regression model
PS-Matched patients with dMMR(n=29)
PS-Matched patients with pMMR (n=29)
