# Supplementary figures and images for: Survival outcomes analysis according to mismatch repair status in locally advanced rectal cancer patients treated with neoadjuvant chemoradiotherapy
Source: Front Oncol. 2022 Aug 8;12:920916. doi: 10.3389/fonc.2022.920916 (PMC9393758; doi:10.3389/fonc.2022.920916)

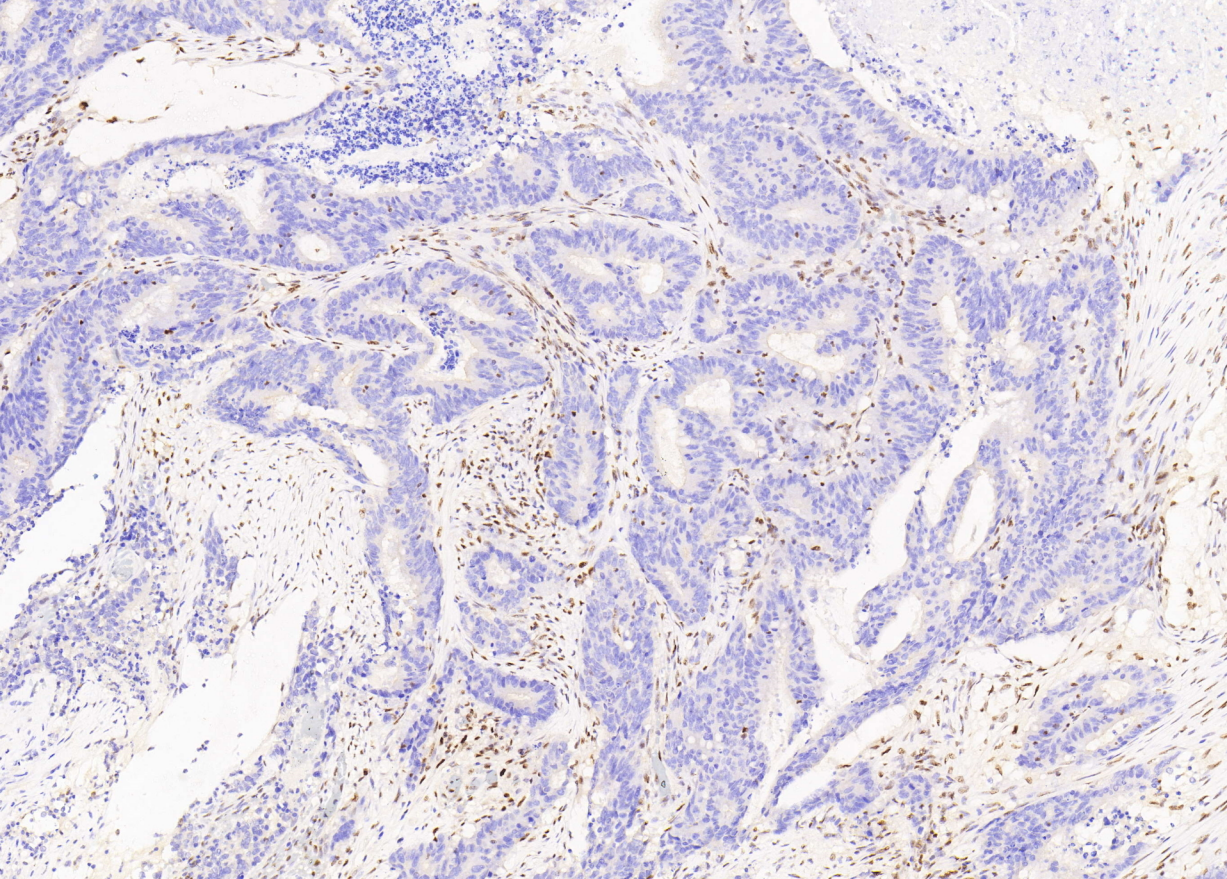

Supplement: Supp Figure 2 — Patient flow chart. MMR, mismatch repair; dMMRdefective MMR, LARC locally advanced rectal cancer; pMMRproficientMMR; PS, propensity score. [file DataSheet_1.zip › Figure 2/Supp Figure 2A-MLH1.pdf]

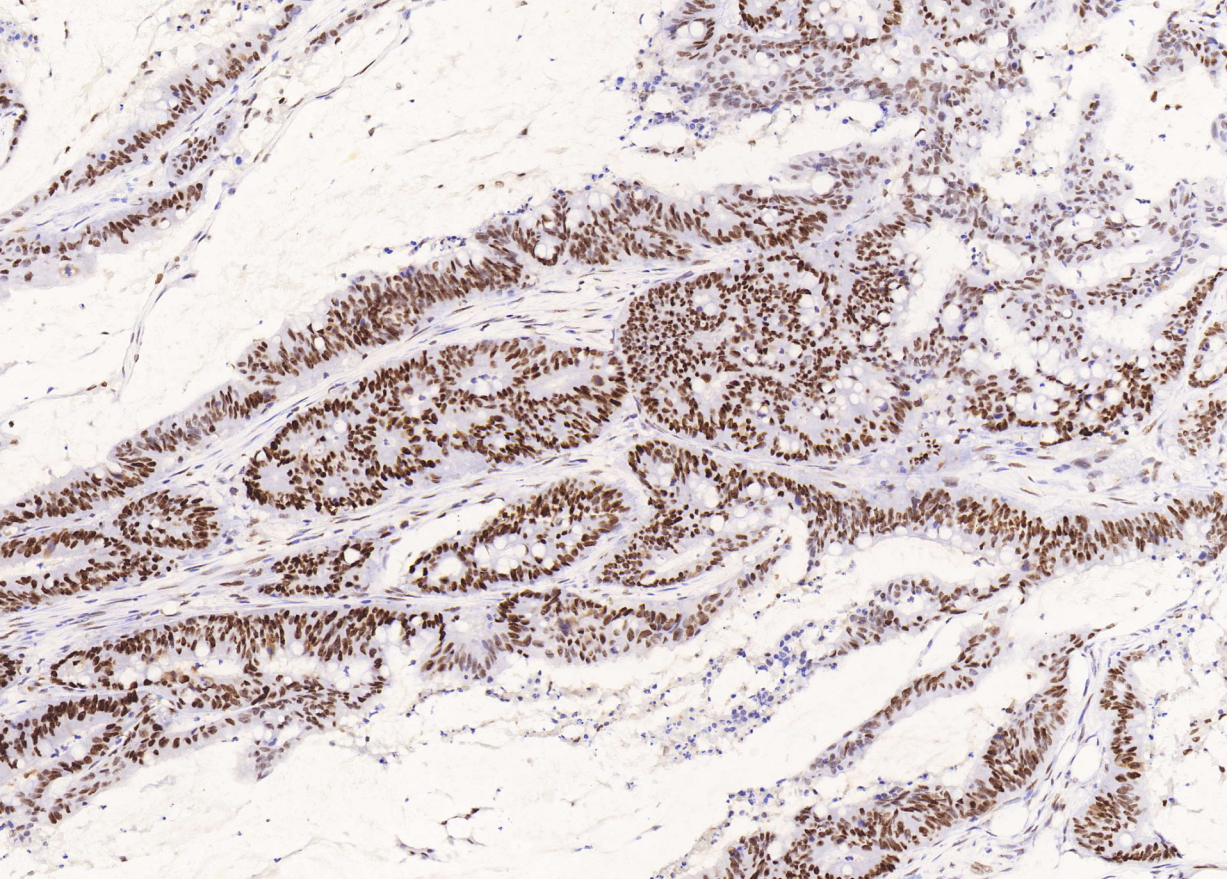

Supplement: Supp Figure 2 — Patient flow chart. MMR, mismatch repair; dMMRdefective MMR, LARC locally advanced rectal cancer; pMMRproficientMMR; PS, propensity score. [file DataSheet_1.zip › Figure 2/Supp Figure 2B-MSH2.pdf]

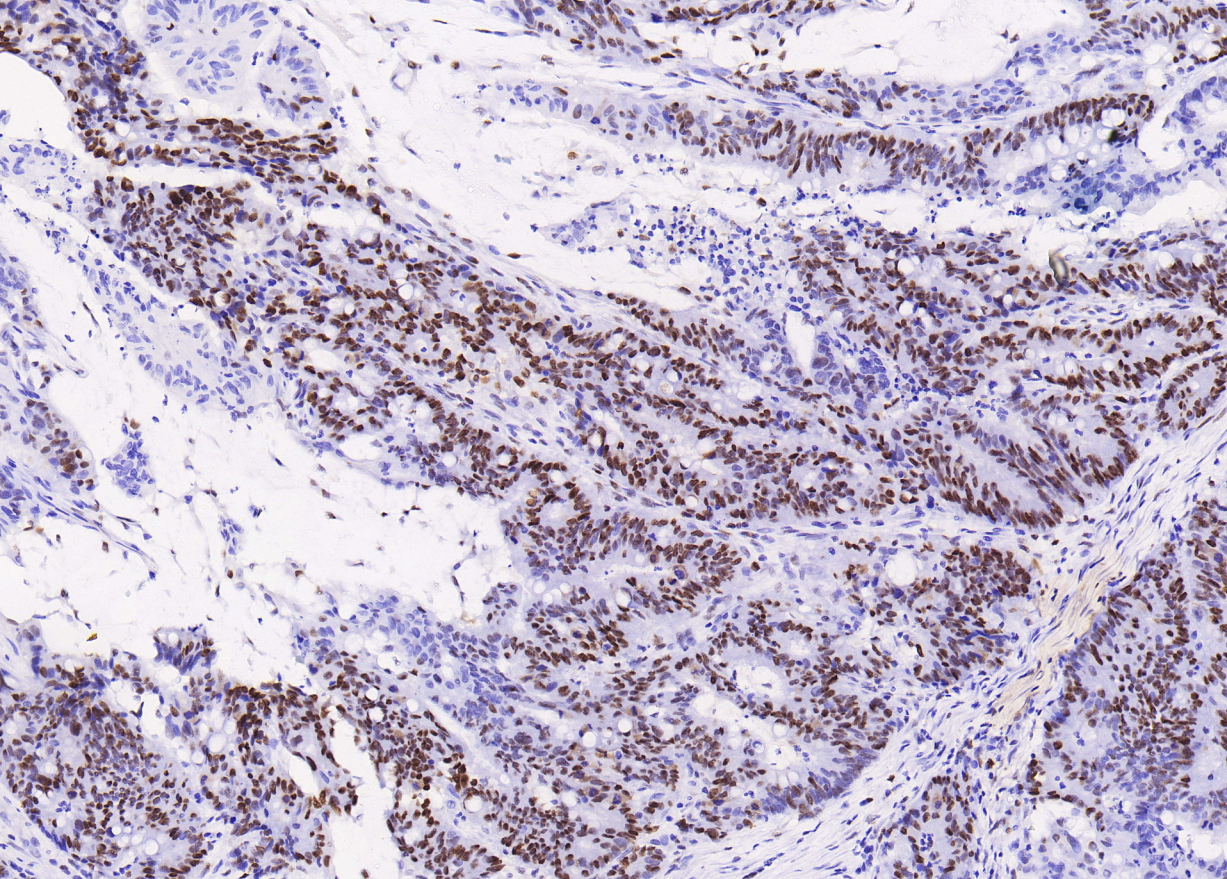

Supplement: Supp Figure 2 — Patient flow chart. MMR, mismatch repair; dMMRdefective MMR, LARC locally advanced rectal cancer; pMMRproficientMMR; PS, propensity score. [file DataSheet_1.zip › Figure 2/Supp Figure 2C-MSH6.pdf]

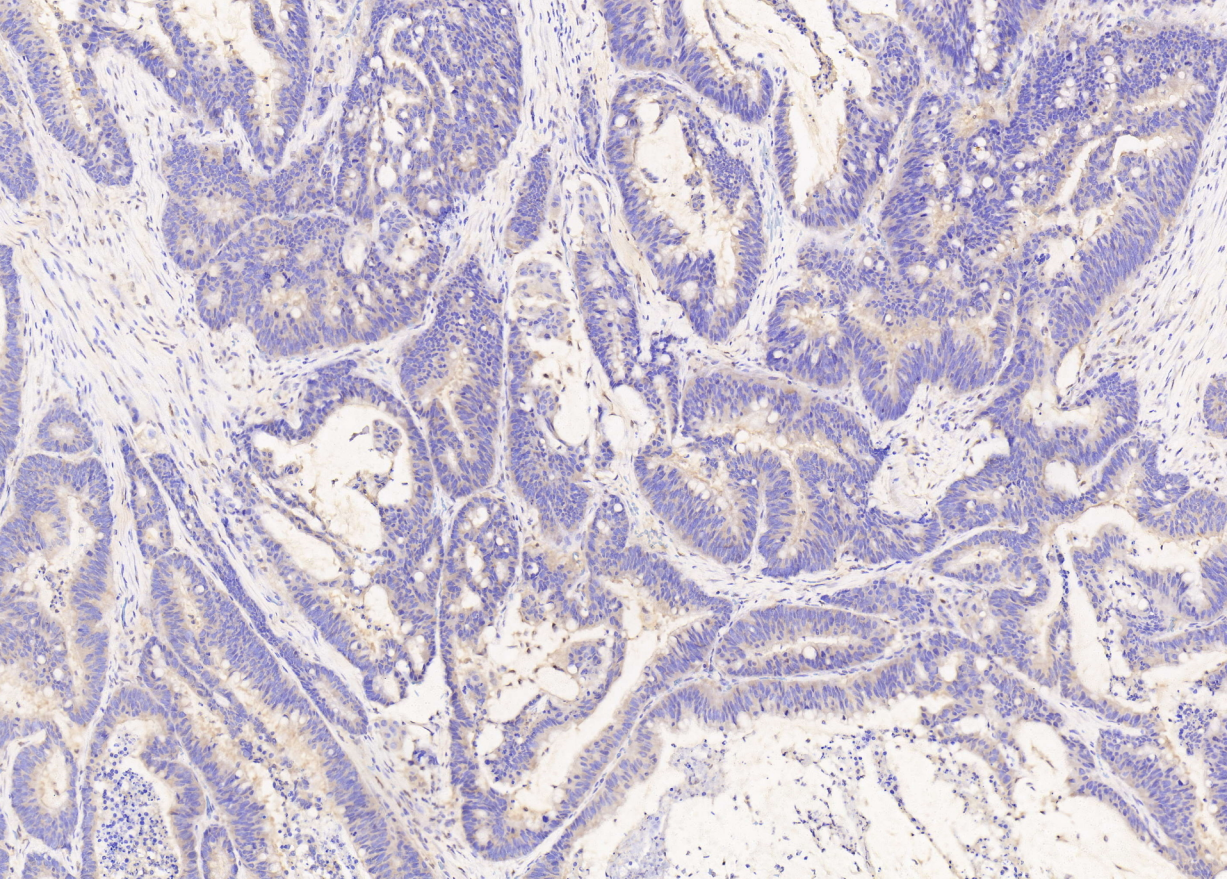

Supplement: Supp Figure 2 — Patient flow chart. MMR, mismatch repair; dMMRdefective MMR, LARC locally advanced rectal cancer; pMMRproficientMMR; PS, propensity score. [file DataSheet_1.zip › Figure 2/Supp Figure 2D-PMS2.pdf]
