# Supplementary material for: Survival outcomes analysis according to mismatch repair status in locally advanced rectal cancer patients treated with neoadjuvant chemoradiotherapy
Source: Front Oncol. 2022 Aug 8;12:920916. doi: 10.3389/fonc.2022.920916 (PMC9393758; doi:10.3389/fonc.2022.920916)
Supplement: Supplementary file 3 [file Table_1.docx]

Supp Table 1: MMR proteins status of 30 dMMR patients

| **MLH1** | **MSH2** | **MSH6** | **PMS2** | **Count (%)** |
| --- | --- | --- | --- | --- |
| + | + | + | - | 6(20.0%) |
| + | + | - | + | 8(26.7%) |
| + | - | + | + | 5(16.7%) |
| + | - | - | + | 6(20.0%) |
| + | - | - | - | 1(3.3%) |
| - | + | + | - | 3(10.0%) |
| - | - | + | - | 1(3.3%) |
|  |  |  |  | **30(100%)** |

Note: “+” denotes presence of MMR proteins and “-” for loss of MMR proteins.
