# Supplementary material for: Survival outcomes analysis according to mismatch repair status in locally advanced rectal cancer patients treated with neoadjuvant chemoradiotherapy
Source: Front Oncol. 2022 Aug 8;12:920916. doi: 10.3389/fonc.2022.920916 (PMC9393758; doi:10.3389/fonc.2022.920916)
Supplement: Supplementary file 4 [file Table_2.docx]

Supp Table 2: Clinical characteristics of 58 MMR matched patients

|  | **dMMR**  **(N=29)** | **pMMR**  **(N=29)** | **P values** |
| --- | --- | --- | --- |
| Age at diagnosis (yrs), median (IQR) | 59.0 (46.0, 65.0) | 52.0(44.0, 60.0) | 0.5250 |
| Male sex, n (%) | 24 (82.8%) | 26 (89.7%) | 0.7057 |
| Weight(kg), mean (SD) | 60.8 (11.12) | 61.4 (10.94) | 0.8136 |
| Body mass index(kg/m^2^), mean (SD) | 21.4 (3.40) | 21.4 (3.79) | 0.9597 |
| Differentiation, n (%) |  |  | 0.8069 |
| Low | 3 (10.3%) | 2 (6.9%) |  |
| Low-middle | 1 (3.4%) | 3 (10.3%) |  |
| Middle | 22 (75.9%) | 23 (79.3%) |  |
| Middle-high | 1 (3.4%) | 0 |  |
| High | 2 (6.9%) | 1 (3.4%) |  |
| Distance from anus to tumor margin(cm), mean (SD) | 4.6 (2.50) | 4.7 (2.08) | 0.8869 |
| Max diameters of tumor(cm), mean (SD) | 3.0 (1.72) | 2.7 (1.38) | 0.3751 |
| CEA before surgery (ng/mL), median (IQR) | 2.8 (2.0, 3.8) | 3.9(2.1, 5.0) | 0.1811 |
| CA19-9 before surgery (U/mL), median (IQR) | 6.5 (3.5, 15.8) | 11.9(4.3, 24.7) | 0.2492 |
| Pathologic type, n (%) |  |  | 1.0000 |
| Tubular | 1 (3.4%) | 1 (3.4%) |  |
| Adenocarcinoma | 28 (96.6%) | 28 (96.6%) |  |
| cT stage, n (%) |  |  | 0.7491 |
| 2 | 5 (17.2%) | 6 (20.7%) |  |
| 3 | 17 (58.6%) | 19 (65.5%) |  |
| 4 | 7 (24.1%) | 4 (13.8%) |  |
| cN stage, n (%) |  |  | 0.7944 |
| 0 | 8 (27.6%) | 7 (24.1%) |  |
| 1 | 10 (34.5%) | 8 (27.6%) |  |
| 2 | 11 (37.9%) | 14 (48.3%) |  |
| cM stage, n (%) |  |  | 1.0000 |
| 0 | 23 (79.3%) | 22 (75.9%) |  |
| 1 | 6 (20.7%) | 7 (24.1%) |  |

Abbreviations: CA, carbohydrate antigen; CEA, carcinoembryonic antigen; dMMR, defective mismatch repair system; IQR, interquartile range; M, metastasis; pMMR, proficient mismatch repair system; SD, standard deviation.
